# Editorial: Mechanisms of Persistence, Survival, and Transmission of Bacterial Foodborne Pathogens in Production Animals

**DOI:** 10.3389/fvets.2018.00139

**Published:** 2018-06-20

**Authors:** Christina L. Swaggerty, Kenneth J. Genovese, Haiqi He, James Allen Byrd Jr., Michael H. Kogut

**Affiliations:** Agricultural Research Service, United States Department of Agriculture, College Station, TX, United States

**Keywords:** persistence, survival, transmission, mechanism, food borne pathogens

Food safety relating to animal commodities is a global matter that directly affects public health and has significant impact on international animal production industries. For years, animal food safety research focused on surveillance and prevalence of foodborne pathogens. But now, studies explore the host-pathogen interface at the molecular, biochemical, and immunological level.

Reducing antibiotic use in food producing animals has steered researchers toward novel ways to break the chain of infection, colonization, persistence, and survival and transmission of foodborne pathogens such as *Salmonella* and *Campylobacter*. Human infections associated with multidrug resistant (MDR) *Salmonella* species in poultry have become a major concern. In attempts to reduce the contamination of poultry products with MDR *Salmonella* species, researchers must characterize these infections in birds, learn how the organisms colonize, establish a “commensal” state, and are transmitted to flock members; and characterize the corresponding host immune response. The study by Liljebjelke et al. looked back at the antimicrobial resistant *Salmonella* serovars recovered from commercial broiler farms in the United States. In this retrospective study, the authors found that the reservoir for antimicrobial resistance remains in the environment; they point to the importance of additional intervention strategies to lessen the future emergence of antimicrobial resistant zoonotic bacteria from these farms. In addition to horizontal transmission of antibiotic-resistant *Salmonella*, the investigators remind us the importance of reducing bacterial loads at the breeder level to reduce future carcass contamination thereby reducing human exposure. In turkeys, researchers have found that a MDR *Salmonella* Heidelberg (*S*. Heidelberg) is easily passed among flock members during the first week-of-age, but cannot establish in the gut of 3-week-old turkeys. In addition, the host response (immune related gene expression) of turkeys to *S*. Heidelberg differs in 3-week-old turkeys vs. those within the first week post-hatch (Bearson et al.). Studies such as this in poultry and other food production species help to shed light on the dynamics of both MDR and susceptible pathogens in these animals and will lead the way to discoveries and implementation of intervention strategies that will ultimately result in the reduction of these pathogens from food production animals and subsequently reduce human infections with foodborne pathogens.

*Salmonella* virulence and subsequent relationships with persistence and survival in the host are firmly associated with colonization and have led researchers down various avenues of investigation including alternative housing of laying hens, the role of biofilms and *Salmonella*, and the signaling pathways associated with increased resistance against *Salmonella* colonization in broilers. With increasing animal welfare concerns and consumer demand, alternative farming practices including laying hens housed in enriched colony cages instead of traditional cage-based housing are being used more and more by the egg industry; however, the consequences of this transition on food safety are not fully understood. Gast et al. monitored fecal shedding of *Salmonella* Enteritidis (*S*. Enteritidis) in hens housed in traditional and enriched cages at various stocking densities and their data suggest *S*. Enteritidis colonization and fecal shedding is negatively impacted by higher stocking density regardless of the cage system. In MacKenzie et al. a review of the formation of biofilms by *Salmonella* spp. and the conditions that cause biofilm formation are compared and contrasted with *Salmonella* spp. that do not form biofilms. *Salmonella* spp. that do form biofilms appear capable of colonizing multiple host species and may “switch” to a more infectious and perhaps more virulent state once inside an acceptable host, a potential adaptation for survival and transmission. In a different approach evaluating *Salmonella* persistence, the study by Swaggerty et al. used an immune peptide array to show that the host kinome profile (protein phosphorylation patterns) in broilers with a high burden of *S*. Enteritidis is distinct from that of broilers with lower levels of colonization. As might be expected, the birds with lower loads of *S*. Enteritidis, meaning the host's immune response has restricted colonization, show increased activity in key signaling pathways associated with chemokine, Jak-Stat, MAPK, and T cell receptor signaling. These findings provide the groundwork for more in-depth studies into specific biomarkers to select individual birds that are more resistant *S*. Enteritidis colonization. Collectively, these studies have laid a solid foundation for future experiments to determine practical approaches to reduce the incidence of foodborne illnesses associated with poultry-acquired *Salmonella*.

Strategies to reduce foodborne pathogens vary widely in both their origins and their effectiveness against colonization and infection of food production species. Investigations into the administration of either pre- or probiotics to feed or water systems has become a hot topic area of research in the pursuit to find alternatives to antibiotics. In Hughes et al. researchers investigate the use of the prebiotic galacto-oligosaccharide (GOS) on host gut microbiota and gene expression and the effects of GOS on *Salmonella* gut colonization. Although GOS did not show significant reductions in *Salmonella* numbers in the gut, GOS did impact gut immune gene expression and the host microbiota compared to control birds and may offer evidence of pathways that may modulate the host response and microbiota toward reducing *Salmonella* colonization of the gut. Hayashi et al. investigated the effects of feeding *Bacillus subtilis* (*B. subtilis*) spores to broiler chickens as a probiotic aimed at *S*. Heidelberg infection and colonization. The researchers found that the *B. subtilis* spores improved performance, had immunomodulatory effects in the gut, altered the gut microflora, and reduced *S*. Heidelberg in the gut. Together, these studies show the potential impact for using pre- and probiotics as alternatives to antibiotics.

*Salmonella* has developed numerous mechanisms to allow it to avoid the host immune response including the ability to survive at various temperatures and develop resistance against short-chained organic acids. Dawoud et al. reviewed the link between thermal resistance and virulence in *Salmonella*. Different animal species have a wide range of body temperature, which can pose as a potential thermal stress challenge for pathogens. One of the host's primary innate immune defenses against microbial infections is merely increasing body temperature. The authors provide an extensive review of regulation of thermal stress response genes in *Salmonella* and indicate the close association between thermal resistance and virulence. One mechanism that *Salmonella* has adapted to be able to persist in a host is to readily survive over a wide range of temperatures due to the efficient expression of the heat (thermal) stress response genes. In a study highlighting another *Salmonella* defense system, Santin et al. examined a strain of *S*. Heidelberg UFPR1 found in broilers in Brazil and found susceptibility to an array of antibiotics and a *Bacillus*-based probiotic but the strain was resistant to short-chain organic acids. Further analysis showed that a comparison between the *S*. Heidelberg UFPR1 genome and the MDR SL476 strain revealed 11 missing genomic fragments and 5 insertions. The deleted genes are involved in cell cycle regulation, virulence, drug resistance, cellular adhesion, and salt efflux, which suggest that these deletions may confer *S*. Heidelberg UFPR1 strain resistance to organic acids and antibiotics. These two studies show how effective *Salmonella* is at evading the host immune response.

*Salmonella* and *Campylobacter* are the two leading causes of bacterial-derived foodborne illness. In the one paper looking at post-harvest intervention strategies, Li et al. looked at *Salmonella* and *Campylobacter* contamination in small-scale mobile poultry-processing units (MPPU). Carcasses treated with commercially available antimicrobials had reduced numbers of recoverable *Campylobacter jejuni* suggesting they are efficacious at reducing this key foodborne pathogen. The authors also demonstrated the importance of raising broilers on clean-shavings, instead of the common practice of using built-up-litter, as a way to also reduce *Salmonella* and *Campylobacter* contamination on MPPU-processed broiler carcasses. These two approaches provide valuable insight into ways to reduce *Salmonella* and *Campylobacter* carcass contamination.

The review by Rothrock et al. shows that in addition to *Salmonella, Campylobacter*, and *Escherichia coli, Listeria* is a major concern within the food animal industry due to their pathogenic potential to cause infection and high rates of mortality. *Listeria monocytogenes* has been isolated from all stages of poultry production/processing and outbreaks have been attributed to poultry. Since live birds are a potential vector for *Listeria* contamination, the authors suggest there is a need for genetic comparison between *Listeria* spp. and *L. monocytogenes* isolated from poultry environments and from other sources to better understand the source of listeriosis outbreaks.

Understanding the complex interplay between food safety and animal production will require a multidisciplinary approach to understand the host-pathogen interaction. This compilation of papers provides examples of the studies that will advance our knowledge and understanding of the mechanisms behind persistence, survival, and transmission of foodborne pathogens in animal agriculture.

## Author contributions

All authors contributed equally to the writing of the Editorial. CS was responsible for compiling each authors contributions into a single document.

### Conflict of interest statement

The authors declare that the research was conducted in the absence of any commercial or financial relationships that could be construed as a potential conflict of interest.

